# Praying until Death: Apostolicism, Delays and Maternal Mortality in Zimbabwe

**DOI:** 10.1371/journal.pone.0160170

**Published:** 2016-08-10

**Authors:** Dodzo Munyaradzi Kenneth, Mhloyi Marvellous, Moyo Stanzia, Dodzo-Masawi Memory

**Affiliations:** 1 Centre for Population Studies, University of Zimbabwe, Harare, Zimbabwe; 2 Institute of Development Studies, National University of Science and Technology, Bulawayo, Zimbabwe; Leibniz Institute for Prevention Research and Epidemiology (BIPS), GERMANY

## Abstract

Religion affects people’s daily lives by solving social problems, although it creates others. Female sexual and reproductive health are among the issues most affected by religion. Apostolic sect members in Zimbabwe have been associated with higher maternal mortality. We explored apostolic beliefs and practices on maternal health using 15 key informant interviews in 5 purposively selected districts of Zimbabwe. Results show that apostolicism promotes high fertility, early marriage, non-use of contraceptives and low or non-use of hospital care. It causes delays in recognizing danger signs, deciding to seek care, reaching and receiving appropriate health care. The existence of a customized spiritual maternal health system demonstrates a huge desire for positive maternal health outcomes among apostolics. We conclude that apostolic beliefs and practices exacerbate delays between onset of maternal complications and receiving help, thus increasing maternal risk. We recommend complementary and adaptive approaches that address the maternal health needs of apostolics in a religiously sensitive manner.

## Introduction

Zimbabwe is constitutionally and predominantly a Christian country. The last few decades saw a massive growth of the Pentecostal charismatic movement and an ultra-conservative apostolic crusade. The previously dominant African traditional religion, characterized by ancestral worship, gradually waned as it was regarded by many as backward, obsolete and ingenuous. Apostolicism is founded on native resistance to foreign divinities preached by the early missionaries. It is therefore characterized by a mixture of Christian and African traditional tenets.

There are many apostolic groupings in Zimbabwe, but the two that command sizable following are the Johanne Marange and Johanne Masowe groupings, aptly named after the founders [1]. These two leaders were both from eastern Zimbabwe and both had deep religious experiences in the early 1930s [2,3]. As a result, both started preaching repentance and baptism among African peoples [4], having followers in Zimbabwe, throughout southern, central, east Africa, and beyond [2]. These groups are collectively known as *vapostori* (the apostolics). Their doctrines are inherently similar but only differ in the degree of radicalism.

An extensive study on apostolics by Maguranyanga [5] revealed that ultra-conservative apostolic sects discourage members from seeking medical care. But similar observations were also made among Catholics on contraception by Mbizvo [6] and Urassa [7]. Most quantitative studies found no relationship between religion and maternal mortality, but instead identified demographic, cultural and socio-economic factors as potential confounders [8,9]. Such conclusions are rather premature, resulting as they do from lack of in-depth and explorative inquiry and failing to see beyond the statistical figures.

It is known that religion abets culture in the formation of beliefs, attitudes and practices. Some traditional practices have been found to be associated with maternal risk [10,11]. Both religion and culture affect the utilization of maternal and new-born health services from a motivational and supply perspective [12,13]. Addai [14] noted that religion influences attitudes and a wide range of behaviors in reproductive health, HIV prevention, and health care utilization.

In Zimbabwe, health, disease and sickness all have spiritual foundations [5,12,13,15]. In fact, the Shona people believe that ancestral spirits influence the health of the living [15]. They are not alone. Most religious people, including Christians, believe that health is a blessing from God and disease a curse from the devil. Apostolic display maternal practices that are significantly different from most other religious groupings [5]. For example, their pregnant women display a higher propensity to deliver outside the formal health system, without skilled attendance [16]. Izugbara [17] noted that non-institutional deliveries by unskilled attendants remain common in much of the developing world, notwithstanding decades of modernization and advances in medical technology. This increases the risk of maternal and neonatal mortality [18,19].

Nearly two-fifths (38%) of women in Zimbabwe identify themselves with an apostolic denomination [16,20]. Amongst these, Maguranyanga [4] found that some reject modern medicine and therapeutics, preferring instead to get services from spiritual birth attendants (SBAs). Here, they are offered prenatal, intrapartum, postnatal and emergency care in the best way a spiritual health system can afford [5,21]. A claim to offer emergency obstetric care (EmOC) from a spiritual perspective sounds fatal, and it may indeed be. It is known that EmOC can reduce maternal mortality [22], particularly when deliveries are conducted by skilled birth attendants in controlled and appropriate conditions [23]. Both of these are absent in spiritual health systems.

The proportion of institutional deliveries in Zimbabwe has systematically fallen from 77% in 1999 to 69% in 2007 65% in 2012 [21,24,25]. In a review of EmOC services in sub-Saharan Africa, Montague [26] found that, although highly effective, the uptake of institutional maternal care remains poor even when widely available. This has been known to cause higher maternal mortality [27,28]. In Zimbabwe NEDICO [29]found religion as the third commonest barrier to health care utilization. How this happens is yet to be fully investigated.

In many developing countries, institutional deliveries remain low and are possibly worsening [26,27]. A number of researchers have demonstrated that apostolic women have a propensity for non-institutional deliveries [5,21,30,31]. Emmanuel [32] found the rates of maternal mortality among apostolics in Marondera district much higher than the provincial and national rates. In the United States, Kaunitz [33] found that religious women who avoided EmOC had 100 times higher mortality than the statewide rates. This demonstrates that the phenomenon is also common in highly developed states. Hove [34] confirmed lower post-natal care attendance among women of the apostolic faith.

Debate continues on what interventions would save more lives, between increasing accessibility to skilled health care [35–37] and making non-institutional deliveries safer [26, 38,39]. Understanding apostolicism, its effect on delays and maternal outcomes may help settle the argument. The three delays model, proposed by Thaddaeus and Maine [40] was adopted in this study. It provides an excellent framework within which to understand religion as a social construct that affects maternal outcomes. It proposes that women die of pregnancy-related causes due to (1) delay in recognizing danger signs and deciding to seek care, (2) delay in reaching an appropriate source of care and (3) delay in obtaining adequate and appropriate treatment. However, the role of apostolicism in shaping the nature and impact of delays in maternal heath has not been adequately investigated. This study hypothesizes a strong association between the two and seeks to close this gap by demonstrating how apostolic beliefs and practices prolong delays, which in turn increase maternal risk.

## Methods

This qualitative study was conducted in 5 rural districts, one from each of 5 provinces. We selected districts according to the prevalence of the apostolic sects. The resultant sample included Beitbridge, Makonde, Marondera, Mutasa and Mt. Darwin. Fifteen key informant interviews (KII) were undertaken using a key informant interview guide. Ten (10) male apostolic sect leaders and five (5) apostolic female spiritual birth attendants (SBAs) were interviewed at places of worship. The male leaders were targeted because of their familiarity with apostolic beliefs and doctrines. The birth attendants were interviewed for their in-depth knowledge of apostolic maternal care practices. Respondents were selected using an opportunistic sampling approach and in some cases, were identified and tracked using the snow-ball technique. The resultant sample had 10 male apostolic sect leaders with ages ranging from 45 to 70 years, and 5 female birth practitioners aged between 35 and 60 years. The sample was confined to rural areas, where the apostolic practices are not diluted by mixed health seeking practices that are common in urban areas. Special arrangements were made with targeted respondents for interviews at their prayer shrines (*kirawa/sowe*) or at home. The researchers conducted the interviews with the help of two assistants, one to take notes and the other to audio-tape the interviews.

### Data Management and Analysis

In all the Mashonaland districts, data were collected using the vernacular Shona language while both the Ndebele and Shona languages were used in Beitbridge district. With the help of assistants, data were captured through extensive note-taking and audio-taping. These data were transcribed and translated and entered into Ethnography software for analysis. From the transcriptions, the authors identified the emerging themes linked to maternal health practices. Through content analysis, the themes were coded manually. In Ethnography, data were sifted through, codes sorted and transcriptions summarised. The output displayed the relationships in form of a tree diagram, where the trunks represented themes while the branches represented the identified issues around a particular theme.

### Ethical Issues

The study was approved by the Medical Research Council of Zimbabwe (Reference Number: MRCZ/B/423). It respected freedom to participate and adhered to research principles pertaining to privacy and confidentiality. Participants in this study provided verbal consent to respond to questions in the key-informant guide and to be audio-taped. Flexibility was provided for them not to respond to questions that they were not comfortable with. Verbal consent was selected, firstly because the explanation for the purpose of and expectations from the interviews was done verbally by the researcher. Secondly, some respondents requested that they did not want the interviews to appear very formal and serious, as would happen if consent forms were written and signed. Verbal consent was therefore audio-taped as provided for and approved by the Medical Research Council of Zimbabwe. It was also explained that participation was voluntary and withdrawal could be done at any time with no negative repercussions.

## Results

The resultant opportunistic sample for this study had a total of 15 respondents, all from selected rural areas. Ten (10) were male apostolic sect leaders, whose ages ranged from 45 to 70 years. None of them had higher than secondary education, with only three of them having actually completed secondary school. Interestingly, these were the youngest and had more liberal views. Although all were married, 3 reported that they had one wife. Five (5) female spiritual birth attendants were interviewed from the same areas as the males. Their ages ranged from 35 to 60 years. Only one of them had completed secondary school. It was not easy to establish their marital status, as asking the question would have compromised completion of interviews. Table 1 below summarizes the demographic characteristics of the sample of spiritual leaders and birth attendants:

**Table 1:** Socio-demographic Characteristics of Respondents.

	Number	Place of Residence	Sex	Reported Ages	Educational Level	Marital Status
a) Apostolic Sect Leaders	10	All rural	All male	Range: 45–70 years	3 completed secondary; 5 completed primary; 2 did not complete primary	All married: 7 polygamous; 3 monogamous
b) Spiritual Birth Attendants (SBAs)	5	All rural	All female	Range: 35–60 years	1 completed secondary; 3 completed primary; 1 did not complete primary	Not established

The following results reflect the relationship between apostolic beliefs and practices, three-delays and maternal outcomes. These emergent themes are presented in broad sections covering general beliefs, followed by practices during pregnancy, labour, delivery and the puerperium.

### General Beliefs Regarding Sexuality, Marriage and Child-bearing

This study revealed that apostolic beliefs influence sexuality, nuptiality and fertility. They also define the differential role of males and females in society. Some of the practices infringe women’s rights and men have higher social status and power to control women’s health and sexuality. This social construction is accepted as a command of God. Consider this typical remark from an apostolic leader in Mutasa:

‘God created Adam first and then Eve. He said women are weaker vessels (*1 Peter 3*:*7*). They should not be given positions of leadership in church and society. Paul said they should keep quiet in the presence of men (*1 Corinthians 14*:*34–35*). In our church women only provide supportive roles like singing, clapping hands and praying’

(Personal Interview of an Apostolic sect leader, Mutasa).

The sentiment above means that male leaders develop church doctrine, oversee its implementation and monitor adherence. Women are confined to less influential supportive roles such as singing and praying, as if to cheerlead the males. In a few liberal sects, women are allowed to exercise higher gifts such as teaching, prophesying and administering healing. Male domination in religious matters therefore spills into other domains of life, including maternal health.

Apostolic sects are not homogeneous, despite similar beliefs. Some read the Christian Bible yet others do not. Those who neither read nor believe the Bible claim dependence on spiritual revelation, an exclusive preserve of male members. The Bible is scorned as a secondary source of God’s mind that is inferior to the direct pronouncements of the spirit. An apostolic leader in Makonde explained:

‘We follow the Old Testament because it talks about the coming of the Holy Spirit. Since the spirit came, there is no need for scriptures. It is like following instructions in a letter that was posted by a person who then arrived before the letter was delivered. You would rather get the instructions from the person than the letter, isn’t it? He who was to come can now talk to us directly. So we are all guided by the Holy Spirit, nothing else’

(Personal Interview of an Apostolic sect leader, Makonde).

Given the domineering role of men in the apostolic sects, the study established that girls are married off at tender ages as low as thirteen years, usually to elderly polygamous men. Asked how they manage the spread of venereal diseases, a sect leader in Mutasa explained:

‘To manage the transmission of diseases in a polygamous marriage, we marry young girls who are virgins. The spirit shows us these virgins in dreams. A man is supposed to have sexual relations with his wives only’

(Personal Interview of an Apostolic sect leader, Mutasa).

The sexual and reproductive rights and choices of young female members are compromised early in life. They cannot choose marriage partners as match-making is a spiritualized matter exclusive to religious seniors. A sect leaders believes that:

‘Marriage is made in heaven and is conceived in the spirit. If you do it in the flesh, it will not last’

(Personal Interview of an Apostolic sect leader, Marondera).

It can be noted that marriage is the acceptable framework for child-bearing but it is associated with harmful practices such as child- and inter-generational marriages. The risk of maternal complications and infection with sexually transmitted diseases, including HIV, is heightened.

Results also show that members of the ultra-conservative apostolic sects are not keen to send their children to school. Girls are especially deprived in this regard as their purpose in life is confined to child-bearing and rearing. Consider a typical remark below by an apostolic sect leader:

‘We do not allow our children, especially girls, to attend school because formal education was brought by colonialists to make us foolish. Schools teach children to be disobedient, rebellious and it gives them too much freedom. Look, all the social problems we see today are a result of western education. We know because everything was prophesied by the spirit’

(Personal Interview of an Apostolic sect leader, Beitbridge).

Apostolic sects that are against formal education and health were formed in the 1960s and have ever since been a political cauldron of colonial resistance. By sheer numbers, they played a major role during the war of liberation and provided spiritual guidance to the course of armed resistance. The political discourse of the country is still highly influenced by this spiritual demographic. Asked if their religious opinions are not against government, one religious leader remarked:

‘The government supports us and we support it too. We are like fish and water. There is nothing wrong with our ways of worship. We teach our boys skills like carpentry, metal-work, brick-molding or tending livestock, while the girls learn how to raise a family. Is this not what the government has now realized by seeking to change the school curriculum to adopt vocational subjects? This spirit of God never errs’

(Personal Interview of an Apostolic sect leader, Mutasa).

Apostolic sect members feel vindicated in their stance since in 2015 the government proposed changes in the school curriculum. This includes a shift from academic subjects to vocational subjects such as carpentry, metal-work and construction. Apostolic members feel that they pioneered this thought.

Asked to explain why polygamy is widely accepted among apostolic sects, an apostolic sect leader bragged:

‘Men carry the seed and God gave it to them in abundance. So they must sow it. Ever wondered why men never stop reproducing even in old-age while women go into menopause? A man should not abdicate his God-given duties’

(Personal Interview of an Apostolic sect leader, Mt. Darwin).

From the above quotation, it appears that high fertility is promoted and also desired. It also hints on a latent desire for better health for women and children.

This study also noted that apostolic women in polygamous unions were responsible for their economic livelihood as well as that of their children and husbands. Using scripture, men abdicate family and household responsibilities. A sect leader explained men’s obligation in marriage as follows:

‘Nothing. The Bible says in the last days many women shall be married to one man, just to have a name and for the removal of shame. They must provide for themselves and the family (Isaiah 4:1)’

(Personal Interview of an Apostolic sect leader, Marondera).

Because socio-economic responsibilities lie on women, they engage in numerous informal activities to fend for their families. An apostolic birth practitioner had this to say:

‘My clients include women who are away from home on business, including those who sell wicker ware and craftworks. These take long journeys from their homes to sell and collect debts from previous sales. Sometimes they have problems with their pregnancies and they come here after we see them in the spirit and make a promise (*chitsidzo*) [sic]’

(Personal Interview of an SBA, Mt. Darwin).

Further, apostolic women have no rights to property. This was explained rhetorically as follows:

‘How can a married woman own property or anything if she herself is owned by her husband? Everything belongs to the man’

(Apostolic sect leader, Mutasa)

In fact, it is men who derive maximum socio-economic and nutritional benefits from the relay of service from the wives. Women are typically servants of their husbands as one SBA in Makonde explained:

‘A woman must do the best for the man, including offering him the best portions of food, doing his laundry and spoiling him like a baby. This way, you are sure to get his heart. We were taught that buttered bread goes to the father/husband (*chingwa chine margarine kuna baba)*’

(Personal Interview of an SBA, Makonde).

Such beliefs mean women suffer nutritional deficiencies on the backdrop of a huge reproductive burden, which can lead to maternal death.

### The Religious Construction of Sickness amongst Apostolics

The study established that some ultra-conservative apostolic sects do not approve of modern drugs and medical sundries. Such beliefs tend to hold women back at home even when a pregnancy-related complication has been identified. This is typically expressed in the sentiment below:

‘Pills, medicines, bandages and medical ointments are all defiled. They have passed through so many hands, you don’t know whose. We don’t use them’

(Personal Interview of an Apostolic sect leader, Makonde).

The religious perception of modern medicine buttresses the first delay which involves making decisions to seek medical attention. Such beliefs are further supported by the belief that going to the clinic or hospital shows lack of faith in God. Religious leaders, who have the privilege of teaching congregants are strong merchants of these beliefs as reflected in the following quote:

‘How do you run to people as if God has failed? Why trust in man more than God? You are saying God cannot help you yet we know he is almighty. If you are strong in your faith you wait for God’

(Personal Interview of an Apostolic sect leader, Mutasa).

In light of dedication to religion displayed by most followers of apostolic sect members, such beliefs are likely to have a huge toll on maternal practices as well.

The interpretation of injury, sickness and pregnancy complications is also very spiritual. Apostolic sect members, perhaps just like many other religious people, believe that everything that happens to a person originates from the spiritual realm. Human health has a spiritual genesis and accidents, injury and illness are spiritually connected. In the same way, complications in pregnancy are a spiritual curse. Note this remark from an apostolic sect leader in Mutasa:

‘God wants us to breed and multiply on earth. That’s a woman’s primary job. Now when that duty comes with pain and stress, it may be God’s judgement for some sins, most likely unfaithfulness or witchcraft’

(Personal Interview of an Apostolic sect leader, Mutasa).

Such interpretations of obstetric complications encourage women not to seek medical intervention. However, such beliefs also keep women in polygamous marriages from engaging in extra-marital relationships, through fear of complications.

This also reflects in the responses prescribed for the manifestation of complications. A simple solution is for the woman to confess her sins as explained below:

‘The gifted members of the church (*vashandiri*) will pray for her forgiveness and the problem will go. If it’s a demon, it will be cast out. If the baby is not moving during delivery, it will then start to move’

(Personal Interview of an SBA, Marondera).

For some sects, in cases where complications persist, referrals are made to a maternity shrine/clinic, which is normally located in a bush or mountain. However, the referral has to be confirmed first in the spirit by the receiving birth attendants. Thus, even within the apostolic maternal health system, delays are encountered.

It also emerged that the people providing maternal health services in the apostolic maternal health care system did not receive any formal training. Whatever skills the SBAs have are believed to be spiritually imparted. This is elaborated by the remark below:

‘Birth attendants (*anambuya nyamukuta*) were trained by the Holy Spirit to conduct deliveries and to help women with problems. Some can even tell women about complications in advance. Prayers are made and holy water and anointed oil are prescribed to avert the complications’

(Personal Interview of an SBA, Mt. Darwin).

Although the experience of SBAs in assisting deliveries is likely to develop with time, the conditions of delivery remain greatly compromised. It was also noted that there is a succession system for the SBAs. Their skills are believed to be transferable to other church members with the help of the Holy Spirit.

‘At church we identify well-behaved girls who are trained for this kind of work. The Spirit guides us in everything. Although we train them, the Holy Spirit imparts them with skills to bring life to earth’

(Personal Interview of an SBA, Mutasa).

We also observed that the establishment and growth of an informal spiritual maternal health system is supported by a ready demand for services, which reaches beyond the church members to the general public. One SBA confirmed:

‘Some people who are not members of our church just come for help, not to worship with us. We do not send them away but refer them to our birth camps’

(Personal Interview of an SBA, Beitbridge).

SBAs thus provide alternative maternal health care beyond the apostolic membership. The motivation for use of spiritual maternal health care services by non-members begs for further interrogation, as it goes beyond the compulsion of religion. Some clients reportedly to use both clinics and SBAs concurrently.

Even among liberal sects that use modern health care, it was revealed that spiritual healing is the first line treatment. This confirms beliefs around the etiology of disease and sickness although it exacerbates the second delay. An SBA explains:

‘When one falls sick we pray for them. Sickness is caused by bad spirits (*mhepo*). If you don’t pray and go straight to the hospital the doctors and nurses will not be able to diagnose the disease. If you go for an operation, you are likely not to recover from the sleep’

(Personal Interview of an SBA, Mt. Darwin).

It is interesting to note that seemingly harmless religious practices like prayer and respect for religious figureheads can result in fatal maternal outcomes through the effects of delays. In the following section we look at the apostolic beliefs and practices during pregnancy.

### Antenatal Beliefs and Practices

Prevention of pregnancy for spacing or limiting fertility is the first gate to avoiding maternal mortality. However, the current study notes that the use of contraception among apostolic sect members is either low or prohibited. High fertility is doctrinally supported and contraception is believed to be contrary to God’s commandment. A sect leader remarked:

‘We do not use contraception because it violates God’s commandments. He desires us to populate the earth and subdue it (Genesis 1:28). Fertility is God’s blessing (Leviticus 26:9). A woman should give birth until all the ova in her womb are finished. Contraceptives dry up the ovaries’

(Personal Interview of an Apostolic sect leader, Beitbridge).

The womb is supposed to be kept intact and contraception is regarded as interference with divine purposes, particularly among the ultra-conservatives. Yet, among the liberal apostolic sects, contraception is tolerated just as the use of health care facilities. However, the desire for high fertility is still evident. A spiritual birth attendant explained as follows:

‘We allow women to use family planning pills but also advise them not to depend on them. A woman may fail to get pregnant again or face difficulties with falling pregnant when she wants to’

(Personal Interview of an SBA, Makonde).

Among the splinter groups that read and believe in the Christian Bible, male members justify polygamy and high fertility as examples set by Old Testament patriarchs such as Abraham, Isaac, Jacob and Israelite kings such as David and Solomon. Further to that they cite appropriate New Testament scriptures in defense of their beliefs. A sect leader had this to say:

‘Jesus himself taught about the need to be fruitful. He actually threatened to cut off every branch that does not bear fruit, while promising to prune every branch that bears fruit so that it will be more fruitful (John 15:1–3). Those who are not being fruitful (bearing many children) will have a short life on earth’

(Personal Interview of an Apostolic sect leader, Mt. Darwin).

Another male apostolic leader supported this doctrinal stance by arguing against contraception by insisting that:

‘You should not interfere with the work of God inside a woman’s body. The womb is a sacred chamber for God to accomplish his purposes and create the people he wants. Just like Mary was told that he will give birth to the Messiah, women are just God’s means of bringing people into this world. By using contraceptives you are fighting against God himself’

(Personal Interview of an Apostolic sect leader, Makonde).

There is therefore a zealous motivation for high fertility among both males and female apostolics. The doctrine makes it clear that doing otherwise is against God’s commandments and is likely to result in God’s judgement.

This study also established that during pregnancy, the religious beliefs of ultra-conservative groups affect pregnant women’s health-seeking behaviour. Instead of utilizing healthcare facilities, pregnant women are instead motivated to depend on the healing power of God through prayer and anointed artefacts. An apostolic sect leader explained:

‘We do not allow pregnant women to go to hospitals or clinics. Instead, we ask them to be prayed for and to use anointed oil, stones, milk or water. Modern medicines are evil. We depend on God, given our direct communion with the Holy Spirit’

(Personal Interview of an Apostolic sect leader, Mutasa).

The study further noted that among apostolics there is no clear plan to refer complicated cases to higher levels of care. One leader of the Johanne Masowe sect explained:

We pray for the sick and pregnant women. We also give them stones, oil, milk and holy water to use at home. When there is no improvement in their health, we leave it in the hands of God Almighty. He knows his plans for everyone’

(Personal Interview of an SBA, Marondera).

From the above quotation, it can be seen that prayer to God is the ultimate source of help with obstetric complications. There is evidence of giving up or premature resignation when desired outcomes are not realized through religious engagements. This bars women from trying other means or accessing help from referral centres.

A feeling of helplessness is also evident among spiritual birth attendants, who provide care as self-styled midwives at spiritual birth camps (*kuchitsidzo*). One such midwife revealed:

‘When complications arise, we pray to God because He is the one who gives life and is also responsible for death. Death is God’s law because He says “I kill and make life, I bring down to the grave and also bring up” (1 Samuel 2:6). He also said a man, who is born of woman, has a short life that is full of turmoil (Job 14:1). So who are we to argue or contradict with God’s plan whenever there is a maternal death?’

(Personal Interview of an SBA, Mutasa).

The above quotation unmasks low self-efficacy amongst apostolic birth practitioners themselves. This, coupled with a stance against referral to clinics or hospitals, exacerbates maternal risk.

The above sentiment is also related to the value attached to a live birth, which is associated with the quality of care offered during pregnancy and delivery. The results show less concern about loss of a pregnancy. Note this remark:

‘A child is a brick … If it breaks, you make another one’

(Personal Interview of an Apostolic sect leader, Mutasa).

The above quotation implies that immediately after loss of pregnancy or neonatal death, a woman can return to fertility. Such practices compound maternal risk.

This study also found that within the apostolic health care system, referrals follow church protocol. A spiritual birth attendant revealed that:

‘The women who come to us for help are referred by the church members and non-members. Those from within the church are referred here by elders. However, non-members hear about the good work we do and come’

(Personal Interview of an SBA, Beitbridge).

The use of prayer and anointed artefacts seems to start very early in the pregnancy as indicated by an apostolic birth attendant below:

‘As soon as a woman realizes she is pregnant, we advise her to come for prayer. In later pregnancy, the Holy Spirit helps us to see inside her womb if the baby is sitting well’

(Personal Interview of an SBA, Mt Darwin).

Preparations for delivery are also surrounded by high levels of spirituality and religious preparations. It makes some sense especially given that no one is sure of what is happening inside the womb. A birth attendant explains:

‘At hospitals they use machines to tell what is inside of a pregnant woman’s body. You are telling me that a machine can see a spirit? It is all guesswork. When you pray to God he tells you everything about the woman and her baby’

(Personal Interview of an SBA, Mt. Darwin).

Motivation to use health care for treatment of the invisible or spiritual beings is very low. It is therefore preferred to give it to God. An SBA explained that:

‘As the woman approaches delivery, we pray for her and give her anointed water to drink and bath. We check size of cervix by inserting a finger into the vagina. Anointed oil is used to lubricate the birth canal and speed up labour’

(Personal Interview of an SBA, Beitbridge).

In the next section, we look at the beliefs and practices surrounding labour and delivery.

### Intra-partum Beliefs and Practices

Awareness of maternal risks such as bleeding increases chances of taking appropriate action. However, in this study, haemorrhage was scripturally interpreted as normal. An apostolic leader remarked:

Blood loss is normal during pregnancy and delivery. In fact, the Bible says that blood is life (Leviticus 17:11, 14). Jesus Christ came to shed His blood so that we may have life (John 10:10). Giving birth should involve loss of blood without which there cannot be life’

(Personal Interview of an SBA, Beitbridge).

It should be noted that the abovementioned belief compromises risk perception and subsequently delays the recognition of danger.

As earlier noted during the antenatal period, there appears to be a low regard for perinatal deaths among some sects. Yet, neonatal health is usually associated with maternal health, just as maternal health is associated with neonatal health. Still-births are believed to be ‘death’ itself. An apostolic leader noted that:

‘When a woman delivers a dead baby at the birth camp, it is actually an answer to prayer. She has expelled death that was in her body. If not expelled, it can cause sickness, bareness, a distended belly (*chimimbamutekwe*) or even death to the woman’

(Personal Interview of an Apostolic sect leader, Marondera).

The place of delivery is usually a birth camp or the SBA’s home, showing that in emergencies, location of delivery attendant determines place of delivery. We also found that place of delivery determines responses to complications, which mainly revolve around prayer and other anointed objects. An SBA explained that:

‘Prayer is the primary force of healing. But we also pray for water, oil and stones for clients to take home with them. These should not be mixed with any medicines from the clinic or from a traditional healer, or they lose healing power’

(Personal Interview of an SBA, Makonde).

When complications arise, sect members are not compelled to evacuate a woman to the clinic or hospital. It is believed that going to a clinic or hospital demonstrates little or no faith in God. A sect leader emphasized:

‘As a spiritual church (*chechi yomweya*) we are always led by the Holy Spirit. Using drugs and medicines is not different from using herbs from traditional healers (*makwenzi*). We don’t mix practices and we never doubt the power of God’

(Personal Interview of an Apostolic sect leader, Mt. Darwin).

Not only that, medical drugs are believed to be defiled. If not inherently accursed, the drug suppliers or original sources are said to be defiled. A sect leader explained below:

‘Medical drugs are defiled and are administered by unspiritual people. Why should you, in such a weak state of health, entrust your life in the hands of people or things you don’t know?’

(Personal Interview of an Apostolic sect leader, Mutasa).

The ultra-conservative apostolics strongly emphasize spiritual purity (*kururama)*, implicating the health system in desecrations. Further, there is lack of trust in the health system, which does not emphasize relationships between service provider and client.

The study also revealed that hospitals and clinics are a last resort when all spiritual interventions fail. In some cases, dying without medical defilements is preferred as it enhances purity for the after-life. This hesitancy to modern medicine spills into post-partum and newborn care practices, where young children are denied healthcare on the basis of parents’ religious beliefs. A spiritual birth attendant explains:

‘We refer a pregnant woman to a clinic or hospital when the spirit confirms darkness over her life (*rima rerufu*). If she comes here with a complication, I swiftly refer her to the nearest clinic or hospital. If she dies at my home or shrine the police will need statements and I avoid that’

(Personal Interview of an SBA, Mt. Darwin).

It is clear that, consulting religious figureheads further prolongs the first and second delays. The above quotation shows the impact of the first delay in deciding to seek care and the second delay in reaching an appropriate facility of care.

The intrapartum services offered by apostolic birth attendants cover both uncomplicated and complicated deliveries. With complicated deliveries, the risk of maternal death is very high as explained below by an SBA:

‘During delivery, I monitor the progress of labour. If it takes too long, I pray for her and rub anointed oil on her abdomen. I can also tie my waistcloth on her abdomen to assist the baby with descent. Delayed delivery is usually caused by spiritual curses. These have to be broken so that the baby can come out’

(Personal Interview of an SBA, Mt. Darwin).

Typical complications are wrongly interpreted, leading to wrong responses. One SBA indicated that:

‘Some women start to fit during labour and delivery because of demons of infertility *(mweya yechirume*). We pray and cast out the demon. Some babies come our buttocks first or one shoulder sticks out without further progress. We help by twisting and pulling baby’.

(SBA, Mutasa)

As observed earlier, the conditions of delivery are highly compromised by the lack of appropriate resources. Clients are requested to bring their own supplies for delivery. An SBA explains:

‘We ask women to bring their own provisions for use during delivery. These include strings to tie the umbilical cord when the baby is born, razor blade to cut the umbilical cord and soap for bathing the baby. They must also bring the baby’s clothes, candles and matches in case the delivery happens during the night’

(Personal Interview of an SBA, Beitbridge).

Conditions of delivery are heavily compromised among those women who cannot afford the provisions. In the next section we look at apostolic beliefs and practices in the post-delivery period.

### Post-partum Beliefs and Practices

After delivery, apostolics believe in the power of prayer to overcome all evil and anti-life forces. It is evident that, just like in any other religion, the main goal of apostolic maternal health care services is life and health for both baby and mother. Although the beliefs and practices do not differ much with those of the antenatal and delivery period, apostolic practices during the post-delivery period shift heavily towards care of the new-born.

Since SBAs also offer post-delivery services, clients feel no obligation to seek expert help from clinics and hospitals. It is worth noting that most of the post-partum services are offered in a culturally and religiously sensitive manner, which probably explains high use by women. An SBA explains:

‘We help the woman with food that quickly strengthens her back and tightens the vagina. A young woman giving birth for the first time needs someone experienced to help her with the baby because she does not know anything’

(Personal Interview of an SBA, Marondera).

Besides material help, women are also given sexual and reproductive health education that is religiously appropriate. Consider the remark below:

‘We advise women to refrain for engaging in sexual intercourse at least for a whole month. This gives them time to recuperate and gain strength. They should also avoid heavy manual labour and stay indoors to protect the baby from breathing bad air and getting sick’

(Personal Interview of an SBA, Makonde).

Worth noting is that some of the advice is consistent with professional advice from clinics and hospitals, although differing in the level of detail. However, in spite of the maternal health care advice given to women, postpartum complications may arise, upon which clients are expected to use anointed water, oil or milk. If the complications persist, they go back to the shrine for prayer.

‘When complications occur after delivery, we encourage women to use the holy water that we give them. They can also come back for prayers and confession of sins’

(Personal Interview of an SBA, Mt. Darwin).

It is however worrying that post-partum haemorrhage can be accepted as being normal or as cleansing process by the body. The remark below was made by a birth practitioner:

‘Bleeding after delivery is normal. But if it persists it may be due to retained dirt in the womb or wounds caused by delivery or plain evil spirits. We pray for her and give her anointed water and oil’

(Personal Interview of an SBA, Marondera).

Bleeding generally seems to be surrounded with numerous religious and cultural inferences. Consider the remark made below by an apostolic birth practitioner:

‘If a woman experienced many problems during her pregnancy, she usually bleeds too much. That is good because she has to take out all the bad blood that was causing her sickness’

(Personal Interview of an SBA, Makonde).

The findings above show that apostolic beliefs and practices tend to keep women away from the modern health care system. The recognition of complications, the spiritual interpretation of the danger signs and the responses heighten maternal risk. In the following section we discuss the foregoing results.

## Discussion

This study demonstrates that indigenous apostolicism in Zimbabwe aims for better health for mothers and children. However, a realization of religious exclusion in health care resulted in the emergence and growth of a parallel spiritual maternal health system, manned by spiritual birth attendants. These SBAs are not skilled [23], a reason why this system is likely to contribute to high maternal mortality. This study demonstrates that the general assertion that apostolic sects refuse health care is incorrect. Quite the opposite, the desire for good health and survival of mothers and infants is evident.

While Maguranyanga [5] highlighted the key maternal risk factors prevalent among apostolics, a new aspect coming from this study is that an over-spiritualization of marriage, conception, maternity, obstetric complications and health almost eliminates the need for modern health care, in preference for spiritual care. Also, the existence of a spiritual maternal health care system causes prolonged delays that precede maternal death [40]. For some, spiritual birth camps are the only source of care, yet these are poorly resourced in human skills, equipment, drugs and medical sundries. This partly explains an increasing trend of community deliveries [20,24,25,27]. Even among liberal apostolics who visit clinics and hospitals, this system creates a detour that prolongs the second delay.

Within this system, the environment and conditions of delivery are not ideal. Perez [30]observed that poor skills and environmental hazards lead to poor results with community deliveries. While it is evident that home deliveries are not safe, the existing skills of SBAs can be harnessed in a collaborative health care system to monitor cases in the community and to refer them to the clinics as proposed by Pasha, et al. [22] and Perez et al. [30].

This study also found that health in general and maternity in particular are highly spiritualized matters, confirming earlier findings [5,12,13,15]. However, this study further reveals that the spiritualization of health and sickness tends to diminish not only women’s feelings of control over their health but also that of SBAs. There is a general feeling that adverse maternal health conditions cannot be moderated or changed by human effort and intervention. In some cases obstetric complications are interpreted as the will of God, thus immolating the desire to seek help. Further, this spiritualization influences delays in recognizing danger signs. Lastly, it mediates or dictates acceptable interventions, most of which take a spiritual dimension and are neither appropriate nor adequate.

Results also show that religious figureheads are always the first port of call for women who experience birth-related complications. They are an additional and significant detour in the chain of health consultations, which prolongs delay in reaching appropriate care. As observed in other studies [22,27,28], emergency obstetric care can reduce maternal mortality. Reference to religious figureheads not only prolongs the time it takes in reaching emergency care services but can also increase fatalities if the religious figureheads do not quickly refer cases to clinic and hospitals.

This study found that although SBAs cannot offer proper EmOC services, they still claim to have answers to women’s problems, which can take a social or spiritual dimension. This also increases the time it takes between onset of complications and reaching an appropriate source of care, if ever the women do. In addition, the uptake of EmOC services depends on client motivation [12,13,26]. However, this study demonstrates that, even with adequate client motivation, apostolicism dichotomizes source of care and takes women away from formal health care, not only members but also non-members.

Generally, apostolic beliefs increase women’s life time risk of maternal mortality. Beliefs around God’s desire for high fertility, low status of women, stance against contraception and non-utilization of health care facilities all heighten maternal risk. In Zimbabwe, similar patterns of high fertility, non-use of contraception and high mortality were also noted among Catholics [6] although not confirmed in Dar-es-Salaam [7]. The immediate results of such beliefs include early marriages and cross-generational marriages, which subsequently result in child-bearing that is *too early*, births that are *too close* apart, *too many* children being born and even *too late* in a woman’s life. These *four-toos* heighten women’s life-time risk of maternal death.

This study also shows that, among apostolics, maternal mortality is one result of skewed gender relations. Their doctrine celebrates male domination of females and disempowerment of girls and women in sexual and reproductive health matters. This compromises the latter’s rights and choices earlier in life and longer into their reproductive lives.

A major problem revealed by this study is low perception of severity of obstetric complications. For example, blood loss is accepted as a normal or self-cleansing process by the body. Slow progress of labour or a delayed stage is spiritually decoded and linked to a woman’s past sins, demons or curses. Indeed, religion shapes world views and influences behaviour confirming earlier observations in this regard [14]. The responses to maternal complications are sub-optimal or outright inappropriate while decisions to seek hospital are either delayed or never made. This confirms earlier observations on low utilization of health care among apostolics [21,29,34].

The general failure to embrace religion in maternal health care is clear since biomedical science is viewed as autonomous from religion. While apostolics think the health system cannot handle spiritual matters, the latter finds some apostolic beliefs very irrational. This study confirmed the latent antipathy and suspicion that persists between the apostolicism and modern health care. Yet, modern practice can leverage on SBAs, a readily accessible community resource that is religiously-sensitive. Unfortunately, apostolic maternal health care is overshadowed by international perspectives, professional knowledge and the unwarranted criticism of non-members. In other regions, the efficacy of similar cadres in improving home-based care, treatment and referral of pregnant women was proved. For as long as the modern science of health care has no place for spirituality, the benefits of the latter cannot be realized.

The fact that SBAs serve both members and non-members of apostolicism means there is something they are doing right, at least in the eyes of the beneficiaries. One plausible explanation is that services are offered free of charge, on a benevolent basis, or it is essentially about accessibility and sensitivity to religious needs. Yet it must be remembered that pregnancy and child birth are highly spiritualized experiences in a woman’s reproductive life. Confining them to biology alone creates problems of acceptance to the spiritually-oriented mind. Clearly, SBAs’ services address these needs and meet the expectations of a spiritual clientele. Is modern health care ready to embrace and fit into a niche that religion already claims? How can this be operationalized?

We acknowledge that the results of this study may be compromised by our reliance on key informants alone. These interviews are susceptible to error, bias and misinterpretation. Had there been other sources of data specific to the selected geographical locations, triangulation would have been preferred. Indeed, the study revealed key insights on the relationship between apostolicism and maternal outcomes but the purposeful sample may not be big enough to generalize for the country. Another flaw we acknowledge in this study is that it did not get the opinions of women who might have received services from SBAs. This is because the study aimed to get expert opinion of apostolic leaders and health practitioners. However, the clients’ voice may have helped to validate the opinions. Again, while our focus on rural areas aimed to give the perceptions of respondents who do not have other easily accessible and affordable alternatives of health care as in urban areas, the study misses a crucial angle. Apostolic practices in urban areas are likely to show new perspectives and dimensions. We recommend further investigations on urban apostolicism, which is more liberal and where the range of choices on maternal health care is wider.

## Conclusion

This study sought to demonstrate how apostolic beliefs and practices cause delays, which in turn indirectly cause maternal death. The findings help to clarify the vague association between the apostolic religion and higher maternal mortality rates hitherto observed in quantitative studies. It aimed to qualitatively reveal the connective risk factors between apostolic beliefs, practices, delays and ultimately adverse maternal outcomes. Results show that apostolicism distorts the recognition of maternal danger signs, underplays severity, lowers self-efficacy, devalues importance of seeking expert help, prolongs delay in reaching a treatment facility and prescribes wrong diagnoses and responses to maternal complications. Comparatively higher maternal mortality among apostolics therefore results from compounded delays. It is clear that apostolicism itself does not cause maternal mortality. However, it tends to work through a web of multiple factors, which themselves cause delays that increase maternal risk. Yet, the emergence of a spiritual health care system provides hope to the problem. It shows an underlying readiness and desire for positive maternal and neonatal outcomes in a system that is sensitive and adaptive. Interventions can harness and catalyse this capacity to complement rather than antagonize the modern health system. Possible strategies for this include partnering with SBAs in maternal health promotion for behavioral and social change. The same cadres can be co-opted as champions for clinic referrals of maternity cases from the community. Lastly, the existing spiritual maternal care services can be made safer by engrafting skilled workers and providing drugs, equipment and medical sundries. However, this should be done with a longer term objective of assimilating religious extremists into the public health system.
